# Supplementary material for: Isolation and Characterization of Key Genes that Promote Flavonoid Accumulation in Purple-leaf Tea (Camellia sinensis L.)
Source: Sci Rep. 2018 Jan 9;8:130. doi: 10.1038/s41598-017-18133-z (PMC5760735; doi:10.1038/s41598-017-18133-z)
Supplement: Supplementary file 1 — Supplementary Data [file 41598_2017_18133_MOESM1_ESM.doc]

**Supplementary Information**

Supplementary Data Figures and Tables

**Isolation and Characterization of Key Genes that Promote Flavonoid Accumulation in Purple-leaf Tea (*****Camellia sinensis* L.)**

Xiujuan He^1,2†^, Xuecheng Zhao^1†^, Liping Gao^1†^, Xingxing Shi^1^, Xinlong Dai^2^, Yajun Liu^1^, Tao Xia^2*^ and Yunsheng Wang^1,2*^

**Figure S1 Summary of differentially expressed unigenes in red-leaf mutants and wild-type tea plants.** Red, Green and blue spots represent up-regulated, down-regulated, no-change unigenes, respectively.


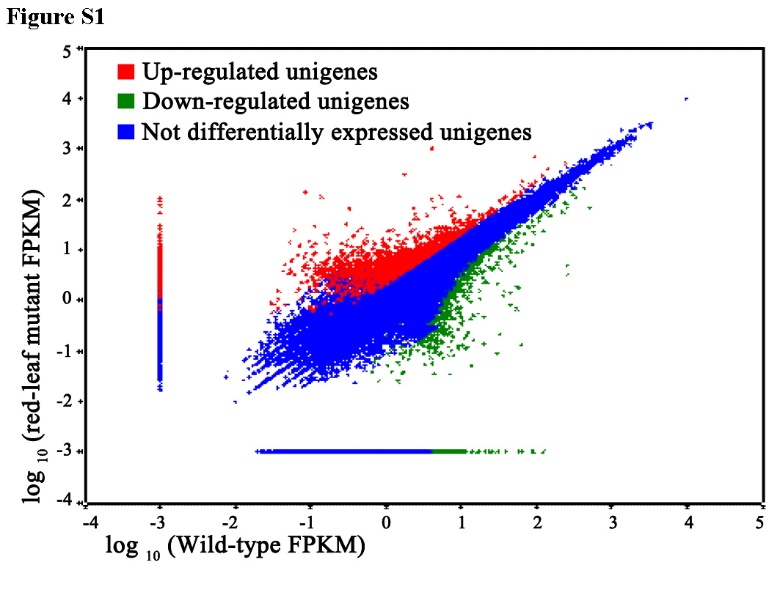


**Figure S2 Subcelleular localization of CsMYB6A protein** (a) Red fluorescence of chloroplast; (b) Green fluorescence of 35S:CsMYB6A-GFP fusion protein; (c) Merged slice of (a) and (b); (c), (e) and (f) highly magnificent slice of white square of (a), (b), and (c).


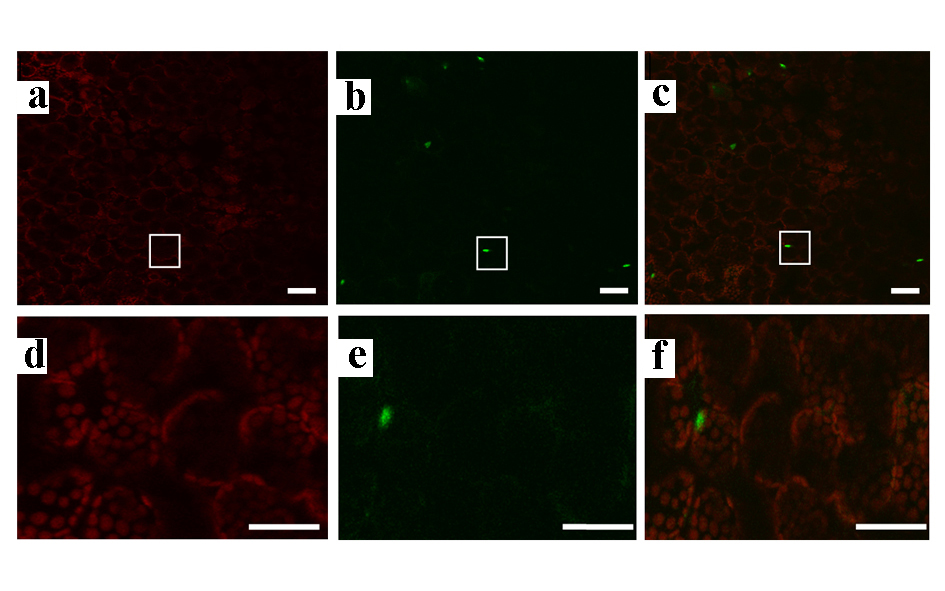


**Figure S3 Effect of temperature and illumination on anthocyanin accumulation in transgenic tobacco overexpressing *CsMYB6A*.** (a) Phenotype of transgenic plants in different conditions; (b) Comparison of total anthocyanin concentrations of transgenic plants in different conditions. (c)Comparison of leaf color of transgenic plants in different illumination conditions.


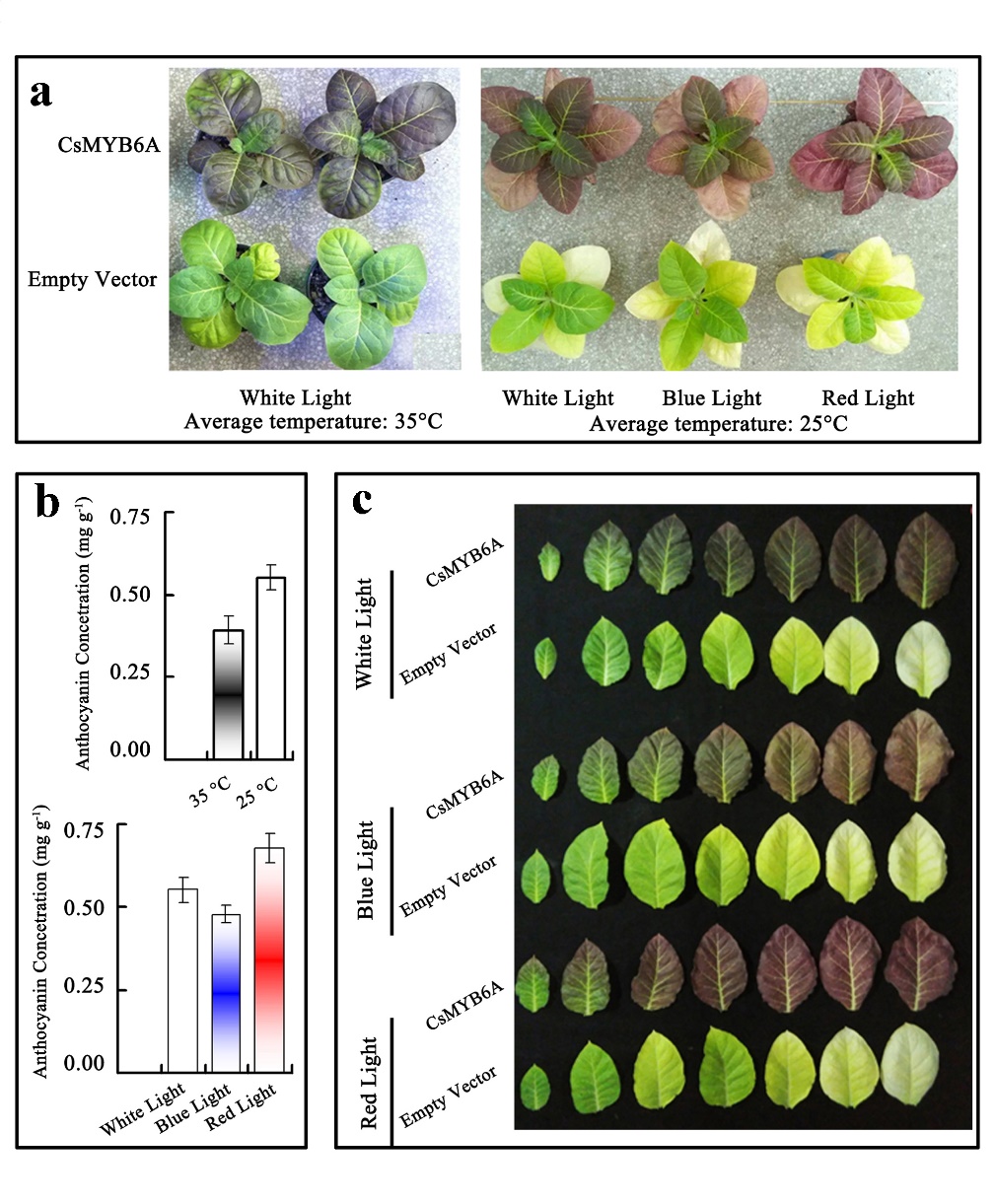


**Table S1 Primers used for cloning, and expression analysis of flavonoid pathway genes**

| Primer name | Primer sequence（5’-3’） Accession number of NCBI | Length of amplicon (bp) |
| --- | --- | --- |
| *CsMYB6A* ORF-PCR | F: ATGGACATTGTTTGTTGTGTTC KX853535  R: TCATTCATCACCTAACAGATC | 765 |
| *CsUGT72AM1 ORF-PCR* | F: 5’-ATGGATAGCTCAAACTCAAAG-3’ KY399734  R: 5’-TTAGTACTTAAGCTTCATCTC-3’ | 1416 |
| *CsMYB6A*-attB | F:GGGGACAAGTTTGTACAAAAAAGCAGGCTATG KX853535  GACATTGTTTGTTGTGTTC  R:GGGGACCACTTTGTACAAGAAAGCTGGGTTCAT  TCATCACCTAACAGATC | 823 |
| *CsPALa*qRT-PCR | F5′-CCGTTCAAGCAAGCAGT-3′ KY615669  R5′-ACATTGTAGCCCTCGTAGA-3′ | 216 |
| *CsPALb*qRT-PCR | F5′-GGGCTGCCATGTTAGTC-3′ KY615673  R5′-TTGATACCCGCGAGGCAGA-3′ | 262 |
| *CsPALc* qRT-PCR | F5′-CAGTGTTAGCCAAGTCGC-3′ KY615671  R5′-GCTCACATTCTTCTCAGCATCG-3′ | 223 |
| *CsPALd qRT-PCR* | F5′-ACCGCCATTGGAAACG-3′ KY615674  R5′-TTCACTCCCTCCCTCGCAGA-3′ | 254 |
| *CsPALe* qRT-PCR | F5′-AGCCGAGTCGCTAAAGG-3′ KY615672  R5′-GCCAACTCCACCTTCAC-3′ | 156 |
| *CsC4Ha* qRT-PCR | F5′-CGATAGAATGGGGCATAGCA-3′ KY615675  R5′-TGGAGGTAGGGGAGTTTGTAGG-3′ | 113 |
| *CsC4Hb* qRT-PCR | F5′-GCTCGGCAGCTATGACATCC-3′ KY615676  R5′-CTCCTCCTACCAACACCGAATG-3′ | 186 |
| *CsC4Hc* qRT-PCR | F5′-GCGATGAAATCTCAACCGTCC-3′ KY615677  R5′-TGACCACAACCTTTGACTCCTTAG-3′ | 198 |
| *Cs4CLa* qRT-PCR | F5′-AGCGGCATAACTATTACCACAGC-3′ KY615680  R5′-TCCCGACGAATAAGGTAGCG-3′ | 279 |
| *Cs4CLc* qRT-PCR | F5′-GGAGGCTAATGAGAGCGAAAT-3′ KY615679  R5′-CATCCACTTGTTGAGGCACG-3′ | 149 |
| *CsCHSa* qRT-PCR | F5′- TGAAGGACCTGCCACGGTTATG -3′ KY615681  R5′- GCCTTATGCTCGCTGTTTGT -3′ | 111 |
| *CsCHSb* qRT-PCR | F5′- GGCAACCCCACCAAACT -3′ KY615683  R5′-CCACCTTATGCTCGCTATTA -3′ | 80 |
| *CsCHSc* qRT-PCR | F5′- TTCCCTTTTCTGGATAGCCC -3′ KY615682  R5′- ACATGTTGCCGTACTCACTTAGC -3′ | 125 |
| *CsCHIb* qRT-PCR | F5′- GTCACAACCACCAAGCCTCTC -3′ KY615686  R5′- GCCCTTCCATTGCTGCAA -3′ | 138 |
| *CsCHIc* qRT-PCR | F5′-AAGCACCAACAAAACCTACCA -3′ KY615685  R5′- AAACACCATTACCAATCGCA -3′ | 86 |
| *CsF3Ha* qRT-PCR | F5′- ACAACAACGCTTACGGCTCTC -3′ KY615688  R5′- AAACCCCCCAACCTTCACA -3′ | 202 |
| *CsF3Hb* qRT-PCR | F5′- TCAAACGCCACACAGACCC -3′ KY615689  R5′- CAACGAAAGCCCCCTCAA -3′ | 126 |
| *CsDFRa* qRT-PCR | F5′- ATTCATCGGCTCGTGGC -3′ KY615690  R5′- TTCTCAGGGTCCTTAGACTCAA -3′ | 246 |
| *CsF3′H* qRT-PCR | F5′- CCGACAGGAGGAGATAGCGATA -3′ KY615695  R5′- GGAAGCAACACCTTGGGGAT -3′ | 277 |
| *CsF3′5′Ha* qRT-PCR | F5′- ATCCTCTCTAAACCCCTCC -3′ KY615696  R5′- ATCCCACAAGTGCCCAT -3′ | 158 |
| *CsF3′5′Hb* qRT-PCR | F5′- CAAAAAAGTCTTAGCCGTCGC -3′ KY615697  R5′- CTCTCAATCCCTTGTAAGTCCATC -3′ | 155 |
| *CsLARa* qRT-PCR | F5′- AAAAGAGGAGGGTGCGG -3′ KY615698  R5′- GGAACTCATCCAAAGGGGG -3′ | 135 |
| *CsLARb* qRT-PCR | F5′- TCAGAGTTTGGACATGACGTGG -3′ KY615700  R5′- CTGACTCCTCTATCAACCTCCG -3′ | 100 |
| *CsLARc* qRT-PCR | F5′- GACAGACGGACGGACAGACA -3′ KY615699  R5′- ACGAGAAGAAAGGTGGGGC -3′ | 158 |
| *CsANRa* qRT-PCR | F5′- GAGTACTTCAAGGCTAAGGGGAT -3′ KY615701  R5′- CAAGCAAACCAAGCAAAACC -3′ | 127 |
| *CsANRb* qRT-PCR | F5′- CTGGCAATCCAAGGAGTGC -3′ KY615702  R5′- GCCCCGTTCCATCAAGC -3′ | 121 |
| *CsANSa* qRT-PCR | F5′- GTTTTCTGCGAACCACCCA -3′ KY615704  R5′- TTTTCCTCCAAGCACCTGAGT -3′ | 150 |
| *NtC4H1* qRT-PCR | F5’-GGGTATCGCCGAGTTAGTCAA-3’ DQ350353.1  R5’-ATCACAGCCTGAAGGTATGGAA-3’ | 132 |
| *Nt4CL1* qRT-PCR | F5’-CAGAAAAGTTGCAGTTGGTCTTAAC-3’ NM_001325625.1  R5’-GCAGCGCAACGACGTCGTCTGGCTG-3’ | 374 |
| *Nt4CL2* qRT-PCR | F5’-CTAAGATCATTGTCACACAAGC-3’ NM_001325625.2  R5’-GTTGAGTGAATAGATATGGAAC-3’ | 353 |
| *NtCHS* qRT-PCR | F5’-AGAAAAGCCTTGTGGAAGCA-3’ XM_009789215.1  R5’-ACTTGGTCCAAAATTGCAGG-3’ | 100 |
| *NtCHI1* qRT-PCR | F5’-GCAGTCTCTTCAAAGTACACGCC-3’ XM_016652447.1  R5’-GAGAGCAATGGAGTCTGTTACCGT-3’ | 171 |
| *NtCHI2* qRT-PCR | F5’-TCCTCCGATCCAGTGA-3’ XM_009588739.2  R5’-CAACGTTGACAACATCAGGC-3’ | 128 |
| *NtF3'H* qRT-PCR | F5’-TCCAAGAATACTGGCCCAAG-3’ XM_009597638  R5’-CTCACAACTCTCGGATGCAA-3’ | 178 |
| *NtF3H* qRT-PCR | F5’-ACAGGGTGAAGTGGTCCAAG-3’ AB289450.1  R5’-CCTTGGTTAAGGCCTCCTTC-3’ | 209 |
| *NtDFR* qRT-PCR | F5’-CAGTTGCTTCCCTTTTCTACCC-3’ NM_001325614  R5’-TGCTTGTCCCTCGGTACTCAGTA-3’ | 118 |
| *NtFLS1* qRT-PCR | F5’-GAACTTGAAGGGAAAAGGGG-3’ DQ435530.1  R5’-TCCCTGTAGGAGGGAGGATT-3’ | 107 |
| *NtLAR* qRT-PCR | F5’-GCAGCAGAAGACTATAGAACTGTG-3’ XM_016641079  R5’-CATGTGTTAGAGCTGCAACTACAC-3’ | 202 |
| *NtANS* qRT-PCR | F5’-AGTGGGTAACGGCAAAGTGTG-3’ AB289447  R5’-CCCTCTGTGAAGAATGCTTTTGT-3’ | 104 |
| *NtANR* qRT-PCR | F5’- CTTGAAGGGTATGCAGATGTT-3’ XM_009632193  R5’-GCAGAGCAAACATATCGTCCAG-3’ | 120 |
| *Nt3GT* qRT-PCR | F5’-GAGTGCATTGGATGCCTTTT-3’ AB723685.1  R5’- CCAGCTCCATTAGGTCCTTG -3’ | 140 |
| *CsGADPH* qRT-PCR | F5’-TTGGCATCGTTGAGGGTCT-3’ XM 002263109  R5’-CAGTGGGAACACGGAAAGC-3’ | 206 |
| *NtActin* qRT-PCR | F5’- TCGGAATGGAAGCTG-3’ AY179605  R5’-TGGTACCACCACTGAGGACA-3’ | 111 |

**Table S2 Summary** [**statistics of unigenes and the corresponding assemblies**](../../../../E:%5C紫芽茶树数据%5Cst转录组数据%5CF14FTSCCKF1363_TEApapT%5CF14FTSCCKF1363_TEApapT%5Cassembly%5Cassembly_statistic.xls)

| **Sample** | **Red-Leaf mutant** | **Wild-type** | **All** |
| --- | --- | --- | --- |
| **Total unigenes** | 85,720 | 82,797 | 77,707 |
| **Total Length of sequence (bp)** | 66,863,572 | 63,389,144 | 78,024,508 |
| **Mean Length of unigenes (bp)** | 780 | 766 | 1004 |
| **Contig N50 (bp)** | 1530 | 1512 | 1675 |
| **Distinct clusters** | 29,525 | 27,649 | 34,191 |
| **Distinct singletons** | 56,195 | 55,148 | 43,516 |
| **GC %** | 44.74 | 44.86 | 44.80 |

Note: Total unigenes represent all the assembled unigenes. Distinct clusters represent the cluster unigenes; Distinct singletons represents unigenes from a single gene.
